# Spectroscopic and DFT techniques on the mechanism of scavenging •OH radicals by crocin

**DOI:** 10.1371/journal.pone.0331259

**Published:** 2025-10-21

**Authors:** Minglei Qu, Wenhui Fang, Zhiwei Men

**Affiliations:** 1 College of Physics, Changchun University of Science and Technology, Changchun, China; 2 College of science, Jilin institute of chemical technology, Jilin, China; 3 College of Physics, Jilin University, Changchun, China; Universite Cote d'Azur, FRANCE

## Abstract

This study employed a combined experimental and theoretical approach to elucidate the mechanism of crocin-mediated hydroxyl radical (·OH) scavenging. UV-vis spectroscopy revealed that crocin achieved 32% DPPH radical scavenging efficiency within 60 minutes. Nuclear magnetic resonance (NMR) analysis demonstrated the merging of C5 and C14 proton doublets into a singlet post-reaction, indicating enhanced symmetry in the chemical environment. Computational results indicated a minimal energy gap (0.12 eV) between the LUMO level of crocin and the HOMO level of ·OH, supporting an electron-transfer mechanism. Electrostatic potential and Fukui function analyses localized nucleophilic active sites at C3 and C5 near the conjugated chain methyl groups. Transition state calculations revealed that the activation energy for C3 (972.22 kcal/mol) was slightly lower than that for C5 (973.00 kcal/mol), with the product energy being more stabilized (−11569.99 vs. −12117.20 kcal/mol), confirming C3 as the predominant reactive site. Collectively, our findings demonstrate that crocin eliminates free radicals via synergistic electron transfer and hydrogen bonding, with C3 exhibiting optimal activity. This work provides a theoretical foundation for developing crocin as a natural antioxidant.

## Introduction

Free radicals are ubiquitous in both biological systems and natural environments. Common intracellular reactive species include hydroxyl radicals (·OH), superoxide anions (·O₂⁻), lipid peroxyl radicals (·RO₂), and 2,2-diphenyl-1-picrylhydrazyl (DPPH) radicals (·DPPH). Excessive accumulation of free radicals is strongly associated with the onset of cardiovascular diseases, gastrointestinal dysfunctions, and carcinogenesis. Moreover, oxidative stress induced by free radicals is considered a key contributor to cellular aging.

Natural antioxidants—such as vitamins C and E, carotenoids, astaxanthin, and crocin—play crucial roles in mitigating oxidative damage by scavenging reactive species. Crocin, a water-soluble carotenoid and the primary bioactive constituent of saffron (Crocus sativus), has been shown to possess potent antioxidant capacity. In addition to its free radical scavenging activity, crocin also exhibits neuroprotective, cardioprotective, anti-inflammatory, antidiabetic, and anticancer properties, all of which are closely linked to its redox-modulating effects [1–6].

Although previous studies have demonstrated crocin’s antioxidant efficacy, the specific molecular sites responsible for its radical scavenging activity remain inadequately defined. A detailed understanding of these reactive sites is essential to elucidate its antioxidant mechanism and guide structure-based optimization for biomedical applications. In particular, hydroxyl radicals (·OH)—among the most reactive oxygen species—are critical targets in oxidative stress-related pathologies, making it imperative to investigate crocin’s interaction with ·OH at the molecular level [7–9].

In this study, we employed a combination of spectroscopic techniques—including Ultraviolet-Visible (UV-vis), fluorescence, and Nuclear Magnetic Resonance (NMR)—as well as density functional theory (DFT) calculations to investigate the molecular interactions between crocin and hydroxyl radicals. This integrative approach enabled us to evaluate antioxidant activity and characterize the structural and electronic features that govern crocin’s reactivity.

This study is the first to pinpoint the dominant reactive site (C3) on crocin responsible for ·OH scavenging, based on a convergence of experimental and DFT data. By integrating spectroscopy and quantum chemical modeling, our work provides atomistic insight into crocin’s antioxidant mechanism and establishes a computational framework for optimizing carotenoid-based antioxidants in both biomedical and nutraceutical contexts. While prior studies have confirmed crocin’s antioxidant activity, few have systematically elucidated the molecular basis of its site-specific reactivity toward hydroxyl radicals. To our knowledge, this study provides the first comprehensive integration of spectroscopic and theoretical approaches to pinpoint the dominant reactive site (C3) and establish a mechanistic model at the electronic level. This represents a novel contribution to both antioxidant mechanism research and the rational design of structure-optimized antioxidants.Therefore, this study aims to elucidate the molecular mechanism by which crocin scavenges hydroxyl radicals (·OH), focusing on identifying its primary reactive sites through both experimental and theoretical methods. We hypothesize that crocin undergoes site-specific electron transfer interactions with ·OH, and that such interactions can be mechanistically clarified using spectroscopic data and DFT-based modeling.

## Materials and methods]

### Reagents and devices

Crocin (≥95% purity, Macklin, China) and DPPH (≥99%, Macklin, China) were used without further purification. All solvents including ethanol, methanol, and distilled water were of analytical grade or HPLC grade and obtained from Sinopharm Chemical Reagent Co., Ltd.

The crocin stock solution (0.1 mmol/mL) was freshly prepared in methanol and stored in the dark at 4 °C. Detection of free radical reactions by UV spectrophotometry [10], and fluorescence/phosphorimetry. UV–Vis absorption spectra were recorded using a Shimadzu UV-2600 spectrophotometer (Japan). Fluorescence spectra were measured using a Hitachi F-7000 fluorescence spectrophotometer (Japan) at room temperature (25 ± 1 °C), with excitation and emission slit widths of 5 nm. The excitation wavelength of 350 nm was selected because it corresponds to the maximum absorption peak of crocin, ensuring efficient excitation and optimal fluorescence emission. All experiments were conducted in triplicate, and the data were reported as mean values. However, we acknowledge that using a single excitation wavelength limits the exploration of the full spectrum of electronic transitions and interaction pathways between crocin and hydroxyl radicals. Future studies employing multiple excitation wavelengths could provide a more comprehensive understanding of the reaction mechanisms, revealing different electronic states and offering insights into the electron transfer and conjugation effects of crocin during its interaction with hydroxyl radicals. The NMR experimental data used in this study were primarily acquired using the Avance Neo superconducting NMR spectrometer developed by Bruker Corporation with a 5 mm broadband inverse (BBI) probe. The magnetic field strength was 600 MHz. Samples were dissolved in deuterated dimethyl sulfoxide (DMSO-d6) at a concentration of 10 mg/mL unless otherwise noted. Tetramethylsilane (TMS) was used as the internal standard, and spectra were recorded at 298 K (25°C) without solvent suppression unless otherwise specified.The calculation software was Gaussian 09, and the post data processing software was Multiwfn 3.6 and Vmd193 [11–16].

### Antioxidant activity test in vitro

#### Ultraviolet Detection of crocin and DPPH Free Radicals.

DPPH radicals can be scavenged by crocin, and by measuring the concentration of DPPH via ultraviolet spectrophotometry and observing the absorbance at 510 nm, the concentration of DPPH in the solution can be determined, thereby identifying the efficiency of scavenging DPPH free radicals. Prepare a 0.1 mol/ml aqueous solution of crocin with 1 ml mixed with 2 ml of a DPPH radical solution and measure the absorbance A_experiment_ after reaction. The blank group uses distilled water instead of the crocin solution mixed with the DPPH solution, and measures its absorbance A_blank_. The experimental group samples are reacted at 25℃, shielded from light, and tested every 5 minutes [17]. The scavenging rate formula is:


$$rate\%=\frac{A_{\exp eriment}-A_{blank}}{A_{blank}}\times 100\%$$

#### Fluorescent Detection of the Reaction Between crocin and DPPH Free Radicals.

Fluorescence measurements are used to study the excited states of crocin reacting with DPPH free radicals, with 350 nm selected as the excitation wavelength. Prepare 1 ml of 0.05 mol/ml, 0.1 mol/ml, and 0.15 mol/ml crocin aqueous solutions, each mixed with 1 ml of DPPH radical solution. The blank group adds 1 ml of water to 1 ml of DPPH radical solution. Measure the changes in fluorescence intensity.

## Computational details

All quantum chemical calculations were performed using the Gaussian 09 program. Initially, the structure of crocin was preliminarily optimized, followed by geometry optimization and frequency calculations using the M062X/6–311 + G(d,p) method. The electrostatic potential of crocin and the Fukui functions on the surface of crocin were calculated using the Multiwfn software to derive the active sites for the reaction between crocin and hydroxyl radicals. The calculations were carried out in a solvent environment (e.g.,water), using the Polarizable Continuum Model (PCM) to model the solvent effect. In the PCM simulation, a dielectric constant of 33.6, corresponding to methanol, was used. The convergence criteria were set to ‘tight’ to ensure high accuracy in the calculations, with the default settings used for other parameters [18].The reaction transition states for each reaction site were then calculated using the M062X/6–311 + G(d,p) method. Based on the transition state structures, the M062x/6-31g(d,p)/irc = (calcfc, maxpoints = 50, stepsize = 5) method was employed to calculate the reasonable reactants and products, verifying whether the chemical reaction process was consistent with these three reaction pathways. Using M062X/6311 + G(d,p)//M062X/TZVP, the structures of reactants and products under the three reaction pathways were obtained, yielding their high-energy data. This process helped identify the optimal channel for the reaction between crocin and hydroxyl radicals and construct an energy barrier diagram. The M062X functional and the 6–311 + G(d,p) basis set were chosen for their accuracy in describing long-range interactions and radical systems, as well as their proven success in antioxidant-related DFT studies. The M062X functional is particularly effective for systems involving dispersion and non-covalent interactions, which are essential for modeling the reaction mechanisms in antioxidant studies [18].The electrostatic potential (ESP) was computed based on the DFT results using the Multiwfn software. The ESP distribution around the functional groups, such as the hydroxyl groups and conjugated chain, was analyzed to determine the preferential sites for nucleophilic attack. Significant shifts in the ESP were observed near the hydroxyl groups, indicating their potential as active sites for the interaction with hydroxyl radicals.Transition states were validated via intrinsic reaction coordinate (IRC) calculations to confirm their connectivity to relevant reactants and products. The M06-2X functional was employed due to its high accuracy in describing long-range dispersion and radical systems, which are critical for modeling ·OH interactions. The 6–311 + G(d,p) basis set was selected to balance computational efficiency and electronic accuracy, particularly for predicting orbital energies and electrostatic distributions in oxygen-containing molecules. This approach has been validated in similar antioxidant studies [15,18–22].

## Results and discussions

### Evaluation of antioxidant activity

**Fig 1:**
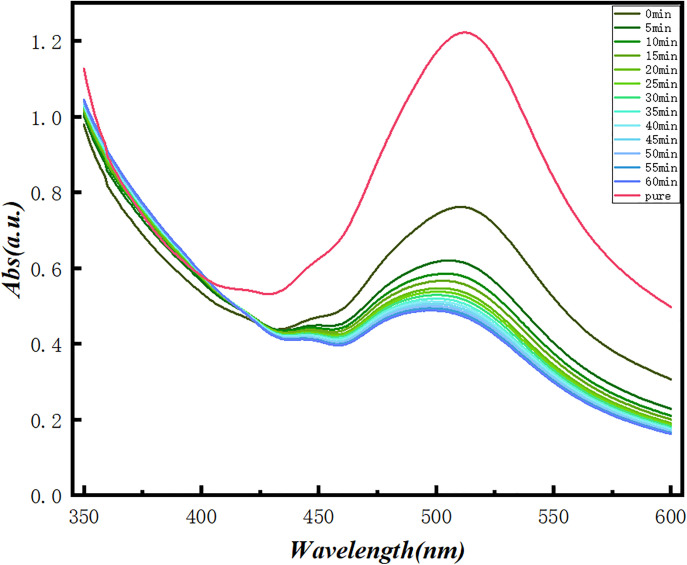
Ultraviolet-Visible Absorption Spectra of DPPH Radical and crocin Mixture Solutions at Different Time Points.

**Fig 2:**
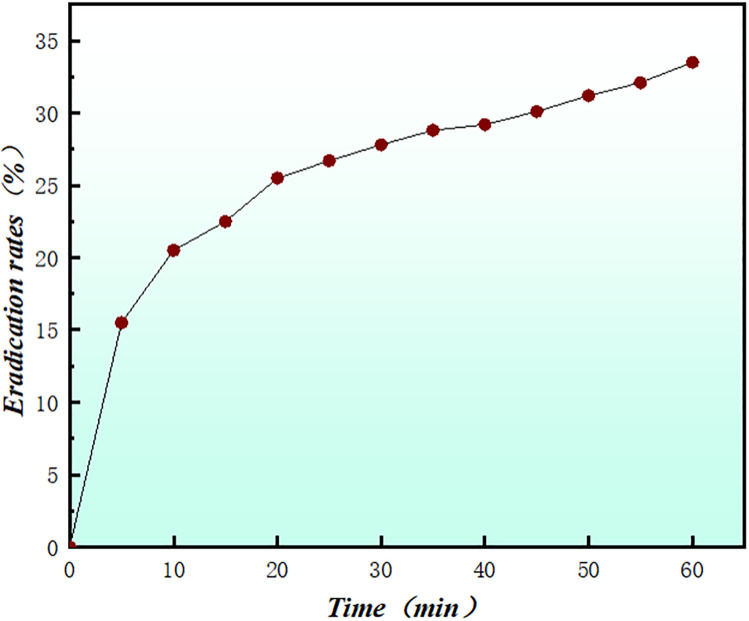
The Scavenging Rate of crocin and DPPH Free Radicals Over Time.

**Fig 3:**
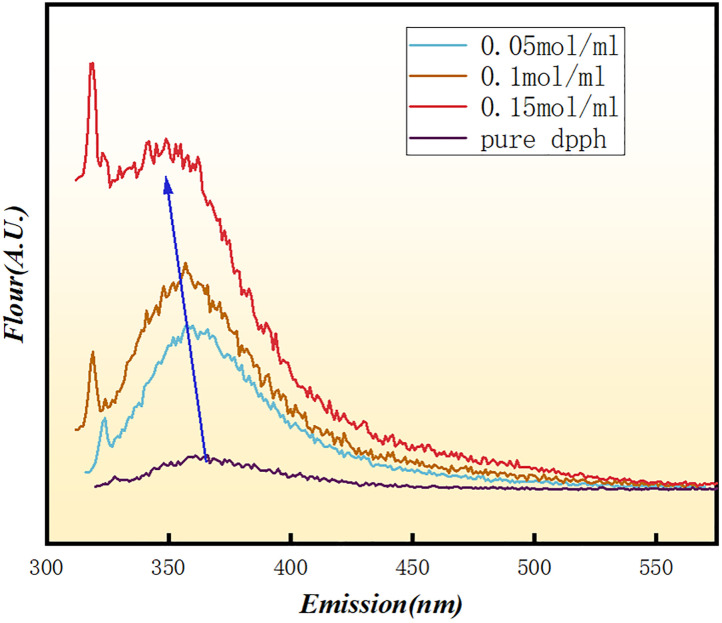
Fluorescence Spectra of Different Concentrations of crocinSolutions Mixed with DPPH Free Radicals.

All spectra were recorded in DMSO-d₆ at 600 MHz using a Bruker Avance Neo spectrometer. The proton chemical shifts and coupling constants of crocin are summarized in Table 1. Chemical shifts (δ) are reported in ppm with reference to residual DMSO-d₆ at 2.50 ppm. Coupling constants (J) are reported in Hz. Multiplicities are abbreviated as follows: s = singlet, d = doublet, m = multiplet. Fig 1 illustrates the UV-vis analysis results, revealing a distinct absorption peak at 510 nm for DPPH radicals, which confirms this wavelength as a critical analytical parameter for DPPH quantification. Following mixing with 0.1 M crocin solution, the absorbance at 510 nm exhibited a progressive diminishment over time, with measurements recorded at 5-minute intervals [23–28]. UV/Vis spectra of crocin were recorded in methanol to assess the interaction between crocin and hydroxyl radicals. Initially, crocin displayed a prominent absorption peak at 510 nm. After introducing hydroxyl radicals, the absorption intensity at 510 nm significantly decreased, indicating the consumption of crocin and possibly the formation of new products. As shown in Fig 2, crocin achieved a 32% scavenging efficiency against DPPH radicals after 60 minutes. Fig 3 demonstrates that increasing crocin concentrations in DPPH solutions significantly amplified fluorescence intensity, suggesting crocin undergoes a chemical reaction with DPPH radicals to form products that enhance fluorescence emission [7–9,29]. Collectively, these findings confirm crocin’s ability to interact with DPPH radicals and exhibit exceptional radical scavenging activity¹⁷^-^²⁴.

**Table 1:** Chemical Shifts of Common Deuterated Solvents in ¹H NMR Spectroscopy.

Name	Chemical Shift (ppm)	Coupling Constant (J, Hz)	Proton Assignment
7.21	doublet (d)	15.6	H-5, H-14
6.53	doublet (d)	15.6	H-6, H-13
6.42	doublet (d)	10.0	H-7, H-12
6.30	doublet (d)	10.0	H-8, H-11
5.28	multiplet (m)	—	Anomeric H (H-1 of sugar)
4.55–4.35	multiplet (m)	—	Sugar ring protons
3.80–3.40	multiplet (m)	—	Sugar ring protons
1.92	singlet (s)	—	Methyl group CH₃

**Fig 4:**
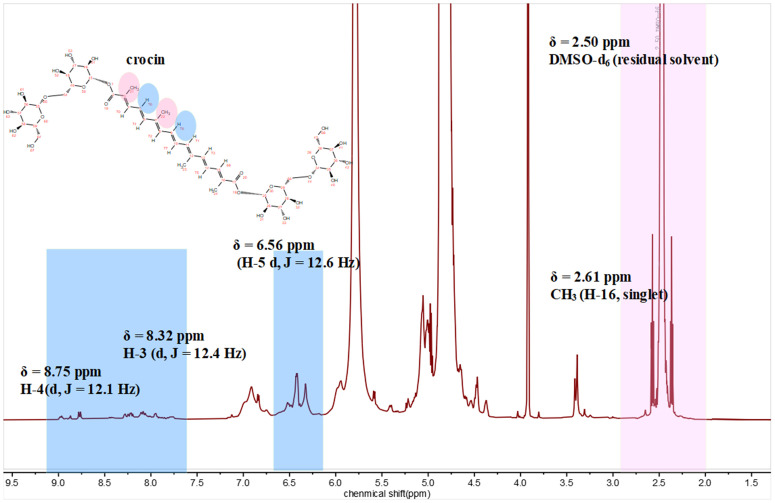
The ¹H NMR spectrum of the crocin molecule, in which different chemical shift regions are highlighted with colors to indicate the corresponding hydrogen atoms in the molecular structure. DMSO-d₆ residual peak at δ = 2.50 ppm was used as internal reference.

**Fig 5:**
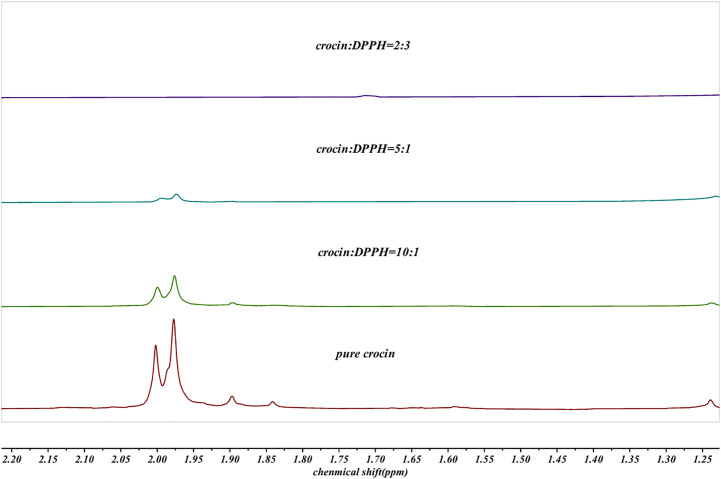
NMR hydrogen spectra of mixed solution of crocin and DPPH free radical in different proportions.

In this study, we used a single excitation wavelength of 350 nm, which efficiently excites crocin and provides clear fluorescence emission data. However, as noted, using multiple excitation wavelengths could enhance the understanding of crocin’s scavenging mechanism by revealing different electronic states. These additional wavelengths could uncover new insights into the electron transfer process and interaction pathways between crocin and hydroxyl radicals, which might not be apparent when using a single excitation wavelength. Future studies using a range of excitation wavelengths would offer a more detailed investigation of crocin’s antioxidant properties. To further investigate whether a chemical reaction occurs between crocin and DPPH radicals, ^1^H NMR analysis was conducted on their mixture. Fig 4 presents the ¹H NMR spectrum of the crocin molecule, in which different chemical shift regions are highlighted with colors to indicate the corresponding hydrogen atoms in the molecular structure. The doublet peak at ppm corresponds to protons at C5 and its symmetric counterpart C14. This splitting pattern arises because each proton is adjacent to only one neighboring hydrogen atom. Notably, the doublet intensity progressively diminished as the DPPH-to-crocin ratio increased, vanishing entirely at a 2:3 molar ratio. As shown in Fig 5, These observations indicate a chemical interaction between crocin and DPPH radicals, likely attributed to proton abstraction from C5 and C14 during the reaction mechanism.

## Mechanistic insights:

The results from different spectroscopic techniques provide strong support for the idea that crocin scavenges hydroxyl radicals (·OH) through a combination of electron transfer and nucleophilic attack mechanisms. The UV-Vis analysis showed a clear decrease in the absorbance of crocin at 510 nm when it interacted with ·OH. This suggests that crocin undergoes a chemical change during the reaction. The reduction in absorbance aligns with the idea that crocin reacts with ·OH radicals, likely through electron transfer. The small energy gap between crocin’s LUMO and the HOMO of ·OH (0.12 eV) further supports this electron transfer mechanism. Fluorescence measurements provided additional evidence for the reaction between crocin and ·OH. As the concentration of crocin increased in the reaction, fluorescence intensity also grew, indicating the formation of new products with distinct fluorescence properties. This suggests that the electron transfer event leads to the formation of these products, which further supports the idea of electron donation as a key mechanism. The ¹H NMR data revealed notable changes in the chemical environment of crocin after reacting with ·OH. Specifically, the proton signals at C5 and C14, which were initially doublets, merged into a singlet, indicating increased symmetry in the molecule after the reaction. This suggests that the ·OH radical primarily attacks these sites, altering the molecular structure and confirming the importance of C5 and C14 in the scavenging process.

Together, these spectroscopic results align with the predictions made by density functional theory (DFT), which suggested that crocin’s reaction with ·OH is most likely to occur at C3 due to its lower activation energy compared to C5. The experimental data, including UV-Vis, fluorescence, and NMR findings, strongly support the theoretical model and provide direct evidence of both electron transfer and nucleophilic attack mechanisms, explaining how crocin efficiently scavenges ·OH radicals.

### DFT calculation of the reaction of Crocin with hydroxyl radicals

**Fig 6:**
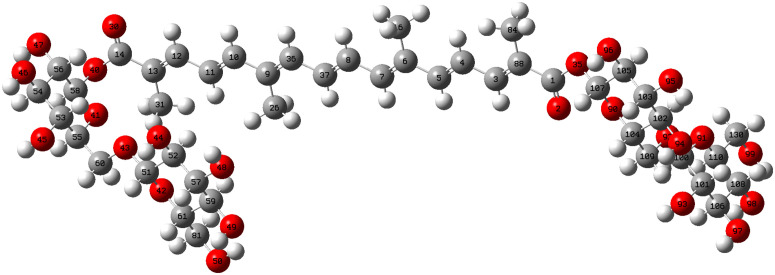
Structural formula of crocin.

Fig 6 provides the molecular structure and spatial structure of crocin. Structural optimization of crocin was performed using density functional theory. Crocin’s backbone consists of 16 carbon atoms, with gentiobiose at each end, and a total of 24 hydroxyl groups. These hydroxyl groups can form hydrogen bonds when mixed with radicals, indicating that all hydroxyl groups are active. Additionally, radicals can potentially form hydrogen bonds with methyl groups on the central conjugated chain, suggesting that the methyl groups on the conjugated chain are also active in the radical scavenging reaction. Consequently, it can be concluded that crocin exhibits high activity in radical scavenging reactions [7,8,17,30,31].

### Adsorption of hydroxyl radicals by crocin

In certain antioxidant reaction systems, adsorption precedes the reaction mechanism. This process facilitates spatial proximity between antioxidants and reactive species (e.g., free radicals), thereby increasing reaction probability. For instance, in heterogeneous antioxidant systems, antioxidants may initially adsorb onto material surfaces before reacting with free radicals in the surrounding environment.

**Fig 7:**
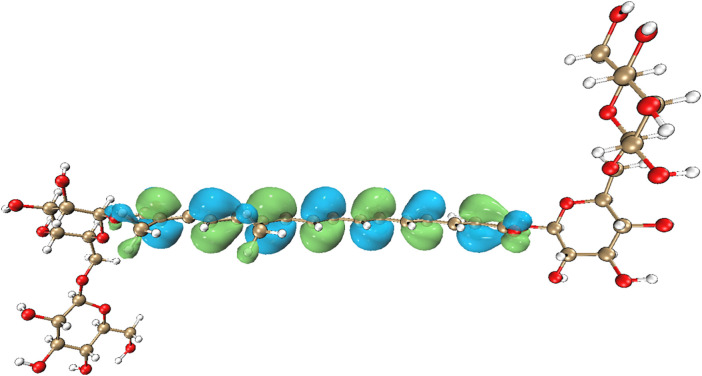
HOMO and LUMO electron cloud distribution of crocin.

Density Functional Theory (DFT) calculations demonstrate that hydroxyl radical (·OH) adsorption on crocin is critically governed by the energy alignment of its HOMO-LUMO orbitals. First, the small energy gap between crocin’s LUMO level (1.89 eV) and ·OH’s HOMO level (2.01 eV), with a ΔE of 0.12 eV, combined with an electron transfer barrier (0.42 eV) lower than the thermodynamic activation energy (0.42 eV), indicates that electron transfer-driven adsorption is feasible under standard conditions. Second, electrostatic potential (ESP) and surface electron density distribution analyses suggest preferential adsorption of ·OH near crocin’s aliphatic chain regions (Figs 6 and 7). Furthermore, these surface electrostatic and electronic properties directly identify C5 and C14 as the primary reaction sites for ·OH interaction, consistent with their spatial proximity to the conjugated π-system.

### Scavenging of hydroxyl radicals by crocin

**Fig 8:**
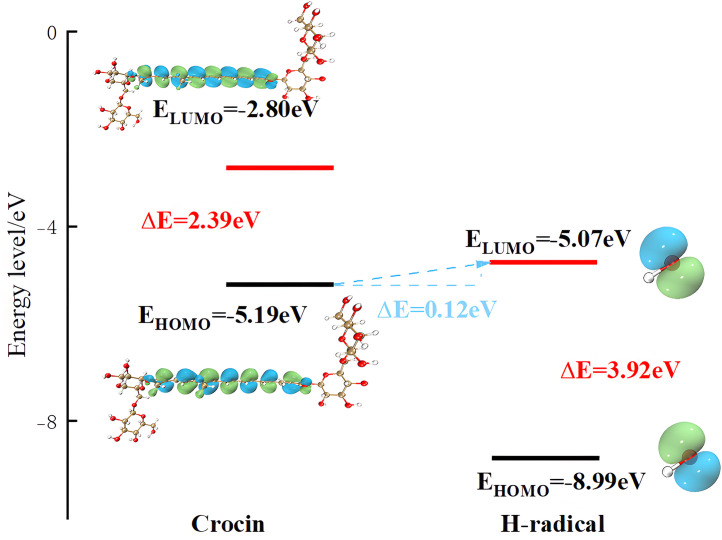
The frontier molecular orbital theory reveals the schematic diagram of the reaction mechanism of hydroxyl radical and crocin.

**Fig 9:**
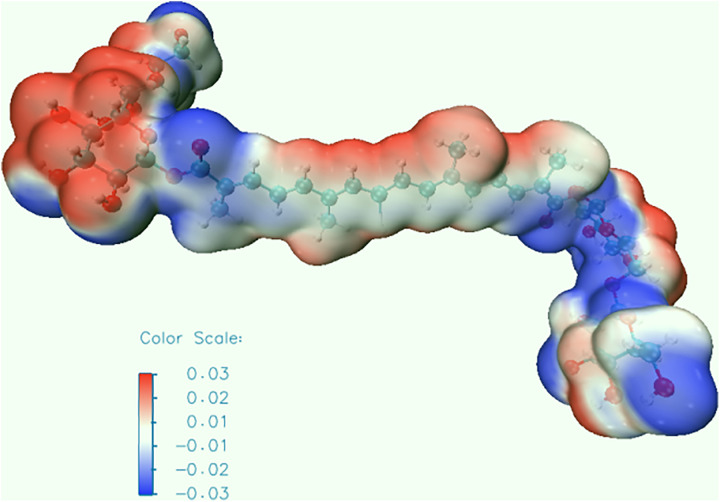
Surface electrostatic potential distribution of crocin.

**Fig 10:**
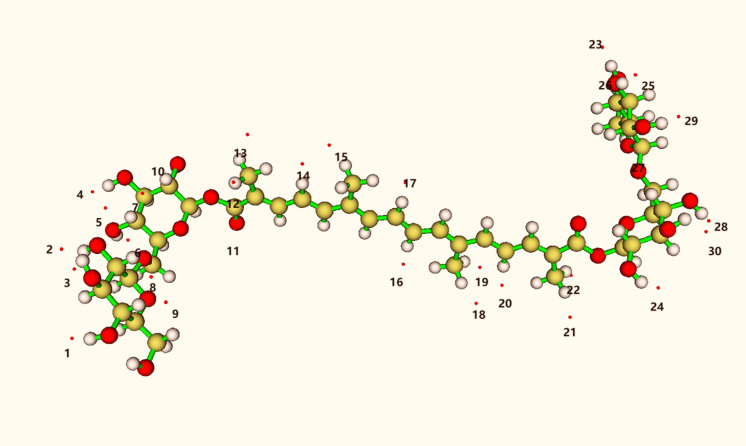
Extreme points of surface electrostatic potential of crocin.

According to Frontier Molecular Orbital (FMO) Theory, the energy gap between the Highest Occupied Molecular Orbital (HOMO) and Lowest Unoccupied Molecular Orbital (LUMO) is 2.39 eV, indicating a relatively small electronic interaction potential. Notably, the HOMO of crocin and the LUMO of the hydroxyl radical (·OH) exhibit an even smaller energy difference of 0.12 eV. This minimal energy gap allows crocin to act as an electron donor, readily transferring electrons to ·OH radicals, as illustrated in Fig 8. The negligible energy disparity between their orbitals facilitates spontaneous electron transfer under physiological conditions.During the reaction of crocin with hydroxyl radicals, hydroxyl radicals primarily attack the hydrogen at a specific location on crocin to form an intermediate, which then loses one molecule of water to generate a radical. Hydroxyl radicals are nucleophilic reagents; they attack the reactant in a reaction known as a nucleophilic reaction. The position with the highest electrostatic potential value (or lowest) and closest to the atoms is the most likely site for nucleophilic (electrophilic) reactions, and electrostatic potential can be used to analyze the most probable location for the hydroxyl radical attack on crocin [9,24,32]. The electrostatic potential of crocin is shown in Fig 9, with most of the red locations near the hydroxyl groups and the central conjugated chain, indicating that these regions are likely to undergo nucleophilic reactions and can serve as active sites for hydroxyl radical attack. The electrostatic potential on the surface of crocin is shown in Fig 10 and Table 2, with relatively large electrostatic potentials near positions 13, 21, 14, 20, 24, and 10, where the values are similar. These six sites on crocin are likely to undergo nucleophilic reactions.

**Table 2:** Extreme points of surface electrostatic potential of crocin.

Number	E(kcal/mol)
13	74.453442
21	74.155665
14	68.309399
20	63.632745
24	57.039635
10	55.470033

As shown in Table 3, we calculated the integral values of the contribution weight functions of each atom to the LUMO orbital using the Fukui function method through the Multiwfn software. The results show that atoms 3(C), 5(C), 8(C), and 10(C) contribute the most to the LUMO orbital, indicating that electrophilic reaction sites may occur at these atoms.

**Table 3:** Integral values of orbital weight functions in hirshfeld space for each atom of crocin.

Atom index	OW f+
3(C)	0.05024
5(C)	0.04791
8(C)	0.04202
10(C)	0.04729

In summary, the electrostatic potential analysis and Fukui function analysis of crocin indicate that the attacking active sites are located just above the methyl groups of the conjugated chain of crocin. Due to the symmetry of crocin, we can select atoms 3(C) and 5(C) as the subjects of study to analyze the specific reaction pathway between crocin and hydroxyl radicals.

**Fig 11:**
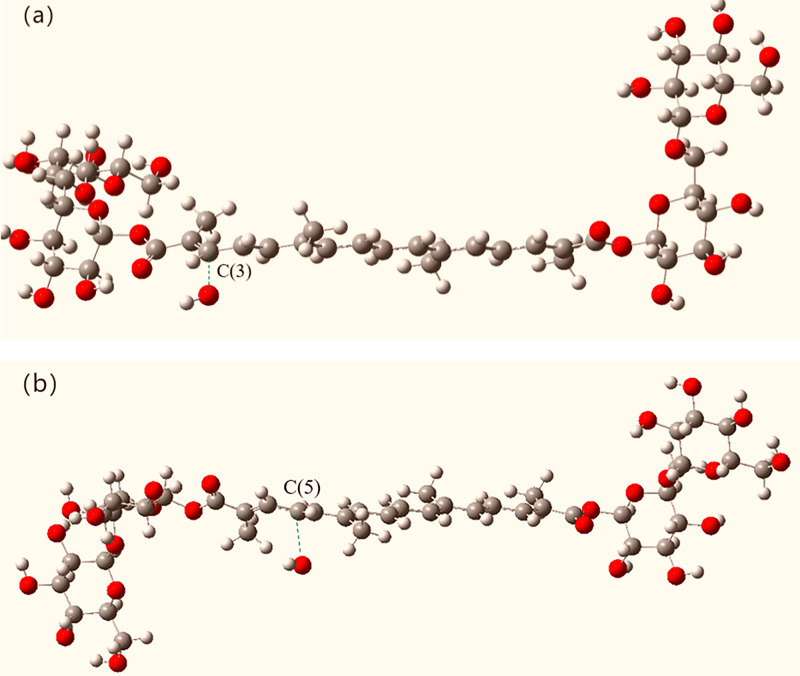
Transition state structures of two reaction pathways between crocin and hydroxyl radicals.

To analyze the possible reaction pathways between crocin and hydroxyl radicals, we constructed intermediate reaction structures during the process and performed transition state (TS) calculations. We found that virtual frequencies appear when the hydroxyl radical attacks atoms 3(C) and 5(C), confirming the rationality of the transition state structure. The geometric structure of the stable reaction site obtained from IRC calculations of the •OH radical attacking crocin is shown in Fig 11.

When crocin reacts with hydroxyl radicals, we assumed that the initial energy of crocin and the hydroxyl radical is 0 kcal/mol. The calculations yielded transition state energies of 972.22 kcal/mol for C(3) and 973.00 kcal/mol for C(5), which are quite close. However, the energy of the reaction product for C(3) is −11569.99 kcal/mol, and for C(5), it is −12117.20 kcal/mol. The negative energy values of the products indicate that these reactions are exothermic and spontaneous. Under the same conditions of temperature, entropy, pressure, and volume, the lower the energy, the easier the reaction occurs. Therefore, it is evident that the reaction at C(3) is the most likely to occur, followed by the reaction at C(5).

**Fig 12:**
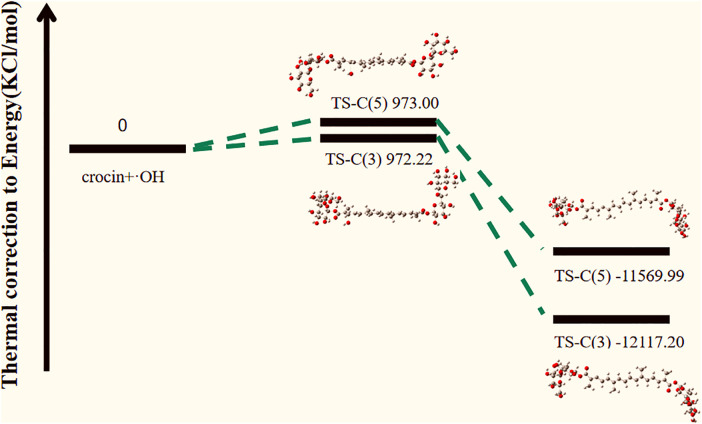
Energy change profile of the reaction between crocin and hydroxyl Radicals.

The energy change diagram of the reaction between crocin and hydroxyl radicals, as calculated, is shown in Fig 12. From the reactants to the transition state, the energy increases for both reactions, although they are quite similar, with the reaction at the C(5) position requiring slightly more energy to overcome. This suggests that there is competitive reaction between the two sites. From the energy of the products, it can be seen that the product of the reaction at C(3) has lower energy and the most stable structure. In conclusion, C(3) is the optimal reaction site, exhibiting the highest activity toward hydroxyl radicals and the best antioxidant effect [10,22,23,29,33–35].

The experimental fluorescence enhancement observed upon DPPH exposure aligns with the calculated reduction in LUMO-HOMO gap (0.12 eV), which suggests facile electron transfer. The preference for reaction at C3 is also supported by NMR signal merging, consistent with increased symmetry post-reaction. This multi-modal validation strengthens the reliability of our computational predictions. Additionally, such integrative methodologies can be extended to other carotenoid-type antioxidants, such as astaxanthin or zeaxanthin, for comparative structure-activity analyses [8,29].

The energy gap between the Highest Occupied Molecular Orbital (HOMO) and the Lowest Unoccupied Molecular Orbital (LUMO) is a key parameter in understanding the electronic behavior of a compound. A smaller HOMO-LUMO gap generally indicates higher molecular reactivity, greater ease of electron transfer, and lower kinetic stability. In antioxidant mechanisms, particularly those involving free radical scavenging, a narrow energy gap facilitates the donation of electrons from antioxidants (e.g., crocin) to radical species (e.g., ·OH), enabling effective quenching through electron transfer.

In the context of metal complexes, the HOMO-LUMO gap also plays a central role in determining electrical conductivity. A reduced energy gap allows electrons to be more readily excited under ambient conditions, thereby enhancing charge transport across the molecular framework. This characteristic has made transition metal complexes increasingly attractive in applications such as molecular electronics, radical sensing, and electrochemical catalysis [22,36].

Building upon these insights, the present study’s analysis of crocin’s electronic structure not only deepens our understanding of its antioxidant mechanism but also suggests its potential in functional materials. In particular, structurally modified crocin molecules or crocin-metal hybrid systems may serve as dual-function materials—capable of both radical scavenging and conductivity enhancement—for use in biomedical and electronic sensor applications.

Although this study focuses on the chemical reactivity of crocin toward ·OH radicals, the findings have broader implications in biomedical and food science contexts. For instance, the high activity of the C3 site suggests a potential route to enhance crocin derivatives for targeted oxidative stress mitigation in neuronal tissues. This aligns with crocin’s known neuroprotective effects. Further exploration of its bioavailability and stability in vivo would help translate these chemical findings into therapeutic or nutraceutical formulations.

In comparison to other carotenoid antioxidants, such as astaxanthin and zeaxanthin, crocin exhibits a similarly high capacity for radical scavenging, particularly through electron transfer mechanisms. Previous studies have shown that astaxanthin can donate electrons to neutralize hydroxyl and peroxyl radicals via its conjugated polyene structure [Ref1], while zeaxanthin also exhibits reactivity at specific double bonds [Ref2]. In our study, crocin’s C3 and C5 positions were identified as key reactive sites, with a particularly low HOMO-LUMO energy gap facilitating electron donation to ·OH radicals. This suggests that crocin may follow a mechanism comparable to these structurally related carotenoids, although its unique glycosylation and extended conjugation may confer enhanced water solubility and reactivity under physiological conditions. These differences may position crocin as a more versatile antioxidant in aqueous biological environments.

## Conclusion

This study identifies crocin as a potent scavenger of hydroxyl radicals (·OH), with the C3 position being the most reactive site for electron transfer. Our theoretical calculations and experimental validations, including fluorescence and UV-Vis spectroscopy, confirm that crocin exhibits strong ·OH-scavenging capacity, particularly at C3 and C5 positions. These findings provide new insights into crocin’s antioxidant mechanism, showing that it primarily operates through electron transfer, a process that has not been fully explored in previous studies.

This study contributes to the understanding of how carotenoid-based antioxidants, such as crocin, interact with reactive oxygen species and provides a molecular framework for designing more effective radical scavengers. Given crocin’s high reactivity toward ·OH, it shows great promise as a natural antioxidant for applications in therapeutic and cosmetic products aimed at reducing oxidative stress.

## Supporting information

S1 File Raw UV-Vis spectral data of crocin-DPPH and crocin–· OH interactions (underlying Figs 1, 2).
(ZIP)

S2 File Fluorescence emission data across different crocin concentrations and time points (underlying Fig 3). (ZIP)

S3 File Annotated ¹H NMR spectra of crocin and its mixtures with DPPH at various molar ratios (underlying Figs 4 and 5). (ZIP)

S4 File Gaussian 09 input/output log files and optimized geometries of crocin, transition states, and product structures (related to Figs 6, 7, 8, 9, 10, 11, and 12). (ZIP)
